# The precarious supply of physical therapists across Canada: exploring national trends in health human resources (1991 to 2005)

**DOI:** 10.1186/1478-4491-5-23

**Published:** 2007-09-25

**Authors:** Michel D Landry, Thomas C Ricketts, Molly C Verrier

**Affiliations:** 1 Department of Physical Therapy, Faculty of Medicine, University of Toronto, Canada; 2 Department of Health Policy and Administration, School of Public Health, University of North Carolina at Chapel Hill, USA; 3 Cecil G. Sheps Centre for Health Services Research, University of North Carolina at Chapel Hill, USA

## Abstract

**Background:**

Health Human Resource (HHR) ratios are one measure of workforce supply, and are often expressed as a ratio in the number of health professionals to a sub-set of the population. In this study, we explore national trends in HHR among physical therapists (PTs) across Canada.

**Methods:**

National population data were combined with provincial databases of registered physical therapists in order to estimate the HHR ratio in 2005, and to establish trends between 1991 and 2005.

**Results:**

The national HHR ratio was 4.3 PTs per 10,000 population in 1991, which increased to 5.0 by 2000. In 2005, the HHR ratios varied widely across jurisdictions; however, we estimate that the national average dropped to 4.8 PTs per 10,000. Although the trend in HHR between 1991 and 2005 suggests positive growth of 11.6%, we have found negative growth of 4.0% in the latter 5-years of this study period.

**Conclusion:**

Demand for rehabilitation services is projected to escalate in the next decade. Identifying benchmarks or targets regarding the optimal number of PTs, along with other health professionals working within inter professional teams, is necessary to establish a stable supply of health providers to meet the emerging rehabilitation and mobility needs of an aging and increasingly complex Canadian population.

## Background

Health Human Resources (HHR) has emerged as a critical factor in health policy planning across Canada [1-5], and within the international community [6-8]. At the federal level, the Pan-Canadian Health Human Resource Strategy acknowledged that, "Appropriate planning and management of HHR are key to developing a health-care workforce that has the right number and mix of health professionals to serve Canadians in all regions of the country" [9]. All provincial and territorial jurisdictions appear to have developed HHR strategies aimed at ensuring that appropriate levels of health providers are in place when and where services are most required [10].

An overall measure of supply within a workforce is the HHR ratio, which is generally expressed as a ratio in the absolute number of health professionals to a sub-set of the population [11]. As noted by Ricketts [12], the origins of the HHR ratio for use in workforce policy can be traced back to work done in the United States where researchers in the 1930s identified a standard of 134.7 physicians per 100,000 (1:742) as a desirable target [13]. This crude HHR ratio included all physicians, and the delineation between primary care, sub-specialty, or even measures of individual or group productivity, had not yet been considered within the estimate. Since that time, the HHR ratios have become benchmarks to measure regional access to health services, and as a method to identify or define an under-serviced area.

In Canada, the published literature has estimated the supply of HHR for larger groups of professionals such as physicians [14-16] and nurses [17-19] across multiple time periods. The literature describing these larger health disciplines is extensive; however very little is known about smaller disciplines such as physical therapists (PTs) and other rehabilitation providers. The emergence of a national focus on inter-professional team practice within primary care and public health initiatives has heightened the need to more fully understand the human resource composition within health care teams.

The Canadian Institute for Health Information (CIHI) published a series of profiles of smaller health disciplines such as audiologists, speech-language pathologists, occupational therapists, and physical therapist [20]; however, these reports did not account for the HHR ratios. The only published study on HHR for PTs was conducted by Landry [21] where the change in the HHR ratio across Canada between 1991 and 2000 was documented. The estimated national HHR ratio was 5.0 PTs per 10,000 population in 2000, which represented a 16.3% increased from 1991[21]. No other peer-reviewed sources concerning physical therapy or other smaller health disciplines were found; however the Canadian Association of Occupation Therapists produced a commissioned report that outlined the need to more fully understand HHR within their discipline [22].

In this current study, we follow up on the initial Landry study [21], and explore trends regarding HHR ratios among PTs in Canada. The purposes of this study were threefold: first, to estimate the 2005 HHR ratio of PTs across provincial jurisdictions by combining population data with lists of registered PTs; second, to compare these findings with those previously reported in order to explore trends over the 15-year period from 1991 to 2005; and third, to interpret the HHR trends from a health policy and workforce planning perspective.

## Methods

The methodology used in this study was identical to the one used by Landry [21]. Briefly, in order to estimate the HHR ratio of PTs in Canada, two sources of data were combined to generate estimates of the ratio of PTs to the population. First, Canadian and provincial population data were obtained from Statistics Canada [23]; and second, the number of registered (active and inactive) PTs was obtained from CIHI [20]. In order to develop a relative HHR indicator, these two data sources were transformed into a ratio of the number of PTs per 10,000 population for the provinces and the country as a whole.

Territorial HHR ratios were not calculated due to lack of valid data regarding the absolute numbers of PTs in the Northwest Territories, Nunavut and Yukon during this particular period. While we recognize that there are very few PTs practicing in the territories, further research must estimate HHR ratios and explore the degree to which the supply of human resources is aligned with demand in these sparsely populated but expansive jurisdictions.

## Results

### Population growth

The population of Canada was 32.6 million in 2005 representing 19.5% growth from 1991, and a 5.9% growth from 2000 [23]. Canada is predicted to experience a population growth of 14% between 2001 and 2021, and the demographics of this growth will include a significant increase in the proportion of the population over the age of 65 years [24]. Though the population of the nation continues to increase, positive growth is not necessarily found across all jurisdictions.

As indicated in Table 1, the eastern-most province of Newfoundland and Labrador demonstrated a negative growth pattern of 9.6% between 1991 and 2005. All other jurisdictions had positive growth during the study period, led by British Columbia (28.0%), Alberta (26.0%) and Ontario (23.1%). However, growth rates in the latter part of this 15-year period represent a different scenario. For instance, in the 5-year period between 2000 and 2005, the provinces of Newfoundland & Labrador, Prince Edward Island, Nova Scotia, New Brunswick and Saskatchewan all demonstrated negative growth ranging from 0.2% to 4.5%. Thus, although the 15-year period from 1991 to 2005 generally show a positive growth pattern in all jurisdictions, trends in population growth in the latter 5 years of this period shows slight negative growth in 5 of 10 provinces.

**Table 1:** Population by province, 1991–2005 [23]

**Province**	**1991 (,000)**	**2000 (,000)**	**2005 (,000)**	**Change (%) 1991–2000**	**Change (%) 2000–2005**	**Change (%) 1991–2005**
Newfoundland and Labrador	568.5	537.9	514.0	-5.4	-4.5	-9.6
Prince Edward Island	129.8	138.3	138.0	+6.5	-0.2	+6.7
Nova Scotia	899.9	942.3	938.2	+4.7	-0.5	+4.3
New Brunswick	723.9	755.6	752.1	+4.4	-0.5	+3.9
Quebec	6,895.9	7,414.7	7,548.6	+7.5	+1.8	+9.5
Ontario	10,084.9	11,894.9	12,416.7	+17.9	+4.4	+23.1
Manitoba	1,091.9	1,149.1	1,170.0	+5.2	+1.8	+7.2
Saskatchewan	988.9	1,017.1	994.9	+2.6	-2.2	+0.6
Alberta	2,545.6	3,059.1	3,207.0	+20.2	+4.8	+26.0
British Columbia	3,282.0	4,101.6	4,203.3	+25.0	+2.5	+28.0
**CANADA**	27,296.8	30,790.8	32,623.5	+12.8	+5.9	+19.5

### Growth in the absolute number of physical therapists

Similar to the overall Canadian population, the absolute number of PTs also grew between 1991 and 2005. According to reports from the CIHI [20], the number of PTs increased from 11,794 in 1991 to 15,772 in 2005, representing a 33.7% growth across the country. Similar to population increase, growth of PTs is not equal across the nation and there are important differences between provinces (Table 2).

**Table 2:** Total number of physical therapists in Canada, 1991 to 2005 [20]

**Province**	**1991**	**2000**	**2005**	**Change (%) 1991–2000**	**Change (%) 2000–2005**	**Change (%) 1991–2005**
Newfoundland and Labrador	121	199	198	+64.5	-0.5	+63.6
Prince Edward Island	32	47	49	+46.9	+4.2	+53.1
Nova Scotia	341	453	529	+32.8	+16.7	+55.1
New Brunswick	240	411	428	+71.3	+4.1	+78.3
Quebec	2,427	3,370	3,677	+38.9	+9.1	+51.5
Ontario	4,509	5,486	5,314	+21.7	-3.1	+17.9
Manitoba	421	556	613	+32.0	+10.3	+45.6
Saskatchewan	337	527	534	+56.4	+1.3	+58.5
Alberta	1,408	1,829	1,924	+29.9	+5.2	+36.6
British Columbia	1,958	2,762	2,506	+41.1	+9.3	+27.9
**CANADA**	11,794	15,640	15,772	+32.6	+0.8	+33.7

As indicated in Table 2, although all ten provinces experienced positive growth in the absolute numbers of PTs from 1991 to 2005, the range was from a low of 17.9% in Ontario, to a high of 78.3% in New Brunswick. However, growth rates between 2000 and 2005 represent a different scenario, and the provinces of Newfoundland & Labrador and Ontario had negative growth of 0.5% and 3.1% respectively.

### Physical therapy health human resource (HHR) ratios

**Figure 1:**
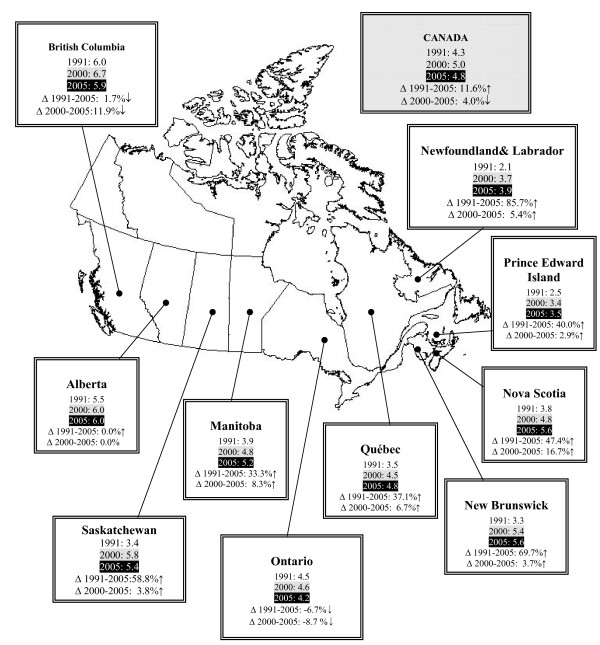
Health human resource (HHR) ratio of physical therapists per 10,000 population across Canada: 1991, 2000 and 2005.

As reported previously, a measure of workforce supply and density is the ratio in the absolute number of health professionals to a sub-set of the population. In order to explore trends over time, the HHR ratios of PTs per 10,000 population in each province were established for 2005, and were then compared to previously reported estimates. Figure 1 is a map of Canada showing the provincial ratios of PTs to 10,000 population estimated at 3 points in time; 1991, 2000 and 2005.

The national average of PTs per 10,000 population was 4.3 in 1991, and 5.0 in 2000. In 2005, the ratio dropped to 4.8. The trend in HHR ratio thus represents an 11.6% growth between 1991 and 2005; however, the data also show a negative growth trend of 4.0% between 2000 and 2005. The relative time period between these three data points limits the degree to which fair and appropriate comparisons can be made; for instance, there is a 10-year period between 1991 and 2000; and a 5-year period between 2000 and 2005. Nevertheless, the latter 5-year period has shown a negative growth trend across Canada.

Figure 1 also outlines that the HHR ratios between 1991 and 2005 increased in almost all provinces. Additionally, most provinces also experienced positive growth (albeit at different rates) between 2000 and 2005. However, the provinces of Ontario and British Columbia had decreased HHR ratio between 2000 and 2005, and Alberta showed no change in across the same 5-year period.

### Change scores regarding population growth and physical therapy HHR ratios

**Figure 2:**
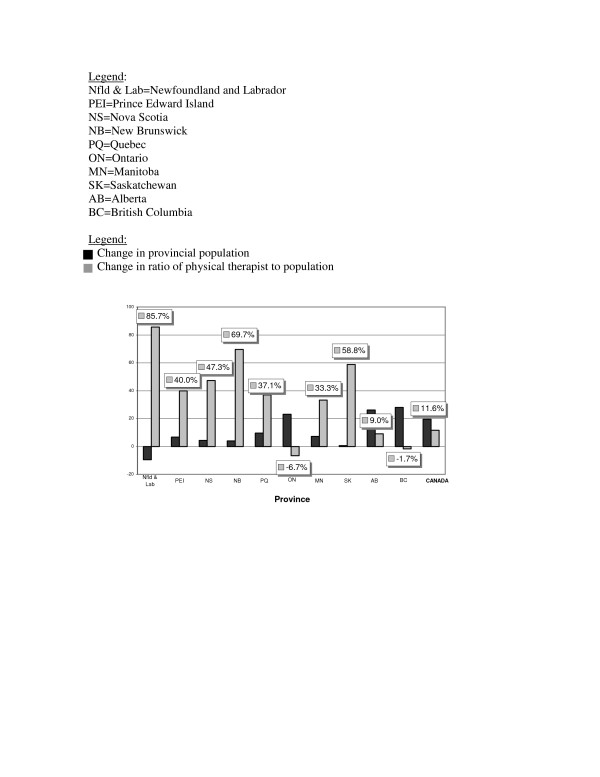
A comparison of the change in population vs the change in the ratio of PTs to 10,000 population: 1991 to 2005.

In order to more fully appreciate the association between trends in overall provincial population growth and physical therapy HHR ratios over time, the change scores of population growth and the change scores of the HHR ratio of PTs to 10,000 population between 1991 and 2005 were plotted in Figure 2.

Figure 2 highlights wide variation between provinces. For instance, Newfoundland & Labrador, the only province to experience a negative population growth during the study period, also showed the greatest change score in the ratio of PTs to 10,000 population with a 85.7% increase between 1991 and 2005. The majority of other provinces, such as Prince Edward Island, Nova Scotia, New Brunswick, Quebec, Manitoba, Saskatchewan and Alberta, experienced increased population growth (albeit at different rates) along with a growth in the HHR ratio of PTs to population. However, in Ontario and British Columbia, positive population growth occurred alongside negative growth in the HHR ratio. In Ontario, a population growth of 23.1% occurred with a decrease in HHR ratio of 6.7%; similarly a population growth of 27.9% in British Columbia occurred along side a 1.7% decrease in HHR ratio.

## Discussion

We have shown that trends in the HHR ratio of PTs to population has increased by 11.6% between 1991 and 2005 (from 4.3 to 4.8 PTs per 10,000), but that the trend in the latter 5 years (between 2000 and 2005) showed a decrease of 4.0% (from 5.0 to 4.8 PTs per 10,000). The reason for this negative growth trend between 2000 and 2005 is not a function of decreasing number of PTs in Canada – indeed, there were more PTs in 2005 than in any other time period. Rather, the trend may partially be explained by the fact that overall population growth appears to be outstripping the growth in the number of PTs across Canada, but especially in Ontario and British Columbia where the greatest proportional population growth occurred during the 15-year period between 1991 and 2005.

The reasons for the limited growth in HHR ratios of PTs in relation to the population are not clear. When comparing these trend data with those data reported by Landry [21], it appears that all provinces other than Ontario and British Columbia have maintained a positive growth in HHR of PTs to population. For instance, Landry reported that Ontario and British Columbia had positive growth of 2.2% and 11.7% respectively; however, when compared with estimates made in this study, HHR ratio have decreased from 2.2 to -6.7 in Ontario, and from 11.7 to -1.7 in British Columbia [21].

As mentioned previously, there were higher absolute numbers of PTs in 2005 than in any previous time period. There are thirteen university training programs across Canada that educates PTs; the majority of these are located in Ontario and Quebec. The five programs in Ontario and the three in Quebec accounted for 65.6% of all graduates in 2004. All thirteen of these educational programs produced 665 PTs in 1995; but by 2004, these programs dropped output by 34 students graduates [20]. Moreover, many of these educational programs have shifted from a bachelor degree to a master entry-level to practice degree during this period of time, which in turn may have contributed to a lowered output of PTs. However, the output of PTs from these education programs appears to have decreased while population growth has increased, and possibly altering the balance between supply and demand for physical therapy services.

The implications and policy interpretation of these findings are complex, and the data collected within this study are not sufficient to establish causation. However critical questions have emerged from this study related to the optimal number of PTs in Canada; for instance, what is the optimal supply of PTs to meet demand across Canada? In other words, is the 2005 national average of 4.8 PTs per 10,000 "high", "low" or "just right"? To our knowledge, there are no needs-based or evidence-based targets or benchmarks regarding the number of PTs per population across settings or conditions. In order to address the question regarding optimal ratios, an analysis of supply and demand for PT is necessary. Demand for health and rehabilitation services are projected to increase in the next decade [24,25]. However, factors that affect this demand have not been fully explored. It is thus critical to develop a forecasting methodology that will estimate demand across settings (i.e. hospital, home and community sectors) and conditions (i.e. arthritis, and other chronic conditions). Once demand for physical therapy services is established, assessing the degree of alignment between supply and demand will become more empirical.

Moreover, further examination of supply-side issues such as individual and group productivity, relative attractiveness of certain sub-sectors for physical therapy practice, episodic balance between the full-time and part-time workforce, along with the emerging shifts form public to private financing of services require in-depth analysis. As supply and demand variables become established, workforce policy and planning research will also become more empirically based, and much less speculative.

## Conclusion

The findings of this study signal a potential disequilibrium between supply and demand within the Canadian physical therapy workforce; however further research is necessary to forecast demand across settings and conditions, and to explore the interaction of complex variables that affect supply. The implication of forecasted population growth, an aging population along with increasing demand and wait times for health service delivery, will require complex policy planning at multiple federal and provincial levels of government. Our results highlight the importance of further examining the precarious balance between supply (i.e. human resources, financing) and demand for health and rehabilitation services, and in establishing targets regarding the optimum HHR ratios. Developing such benchmarks is a first step to establishing a stable supply of PTs which in turn will ensure that clients have access to necessary services when and where they are most needed.

## Competing interests

The author(s) declare that they have no competing interests.

## Authors' contributions

Michel D. Landry designed the study, participated in the data collection, analyzed the data, and wrote successive drafts of the manuscript. Thomas C. Ricketts and Molly C. Verrier both participated in the design of the study, and reviewed successive drafts of the manuscript. All authors have read and approved the final manuscript.
